# The relationship between earthquake-induced post-traumatic stress disorder and breastfeeding attitude and behavior

**DOI:** 10.1186/s12888-024-05803-2

**Published:** 2024-05-07

**Authors:** Erdoğan Öz, Osman Küçükkelepçe, Osman Kurt, Yaşar Kapıcı

**Affiliations:** 1 Adıyaman Provincial Health Directorate, Adıyaman, Türkiye; 2 Faculty of Medicine, Department of Psychiatry, Adıyaman University, Adıyaman, Türkiye

**Keywords:** Post-traumatic stress disorder, Breastfeeding, Breast milk, Earthquake, Maternal mental health, Disaster

## Abstract

**Background:**

This study aimed to investigate the prevalence and severity of post-traumatic stress disorder (PTSD) and analyze the relationship between PTSD and breastfeeding attitudes and behaviors among breastfeeding mothers and women with children aged 0–24 months, all of whom had experienced the earthquake.

**Methods:**

In this cross-sectional survey, a face-to-face questionnaire was administered to 173 earthquake survivors in Adıyaman, Turkey, during June and July 2023. The PTSD Checklist-Civilian scale was used to assess the presence of PTSD, while the Breastfeeding Attitudes of the Evaluation Scale (BAES) was employed to evaluate breastfeeding behaviors in mothers.

**Results:**

Significantly higher PTSD scores (47.6 ± 17.4) were found among women staying in tents, while lower scores (37.0 ± 16.4) were observed in those who continued breastfeeding. 78.6% of women reported decreased breast milk because of the earthquake. Mothers with reduced milk supply had higher PTSD scores (46.1 ± 17.3). Breastfeeding training was associated with higher BAES scores (106.8 ± 56.8) and lower PTSD scores (32.5 ± 11.0). A significant negative correlation was observed between the PTSD score and BAES (*r* = -0.742; *p* < 0.001).

**Conclusions:**

The study demonstrated that breastfeeding may protect mothers against PTSD in the aftermath of earthquakes, emphasizing the importance of breastfeeding education. The higher frequency and severity of PTSD observed among earthquake survivor mothers residing in tents underscores the importance of promptly transitioning to permanent housing after the earthquake.

## Background

After an earthquake, pre-existing psychiatric disorders can intensify, and new conditions PTSD may emerge [1]. PTSD is a significant psychiatric disorder that arises following physical or environmental trauma. Although it can appear at any age, it is more common among young adults, with a higher lifetime prevalence among women than men (0.5%, 1–3%). Its occurrence reaches around 3% in natural disasters such as earthquakes, fires, and floods [1, 2].

Individuals with PTSD experience fear and helplessness due to death or the threat of death, severe injury, or physical harm to themselves or those around them, often resulting in a diminished quality of life which can sometimes become chronic. Common symptoms include insomnia, nightmares, recurring memories of the traumatic event, persistent alertness, and a constant unease related to the fear of reliving the event [3]. Symptoms usually emerge in the days immediately following the trauma and often resolve spontaneously within a few weeks without intervention. However, in some cases, symptoms may persist for months or even years, with onset sometimes occurring months after the traumatic incident [4].

Psychiatric disorders such as depression and anxiety are less common in mothers who solely breastfeed their babies and breastfeed for longer periods [5]. As breastfeeding may protect the mother against PTSD, the risk of PTSD increases in mothers who do not start or cease breastfeeding [6]. However, postpartum depression and PTSD can lead to lower breastfeeding rates. Even though there are favorable data, the relationship between PTSD and breastfeeding has not been exactly established [5].

As is well known during the initial six months of life, breast milk comprehensively meets the nutritional needs of infants. Its diverse composition safeguards infants against diseases and protects mothers against certain cancers. Longer breastfeeding duration positively impacts maternal mental health and infant development because of the unique bond between mother and child [7]. However, natural disasters such as earthquakes significantly disrupt the feeding practices of infants and young children. Despite well-established evidence supporting the uninterrupted continuation of breastfeeding, recommended dietary practices often falter during times of disaster. This neglect jeopardizes the well-being of both mother and child [8].

Concerns about inadequate breast milk during crises, the breakdown of social structures, and unregulated formula distribution can negatively affect nutritional practices. A decline in maternal nutrition following an earthquake and concerns that maternal stress may compromise milk quality may lead to shifts in maternal attitudes and behaviors toward breastfeeding. Consequently, mothers might prematurely introduce complementary foods or discontinue breastfeeding altogether [9].

Based on the question, “What is the relationship between the presence and severity of PTSD and breastfeeding habits in breastfeeding mothers after natural disasters?’’ this study aimed to investigate the presence and severity of PTSD among breastfeeding mothers and women with children aged 0–24 months in the Adıyaman province of Turkey following the earthquake of February 6, 2023. Additionally, the study aimed to explore the relationship between PTSD and breastfeeding attitudes and behaviors in these women.

## Methods

### Study design

This cross-sectional survey was conducted in Adıyaman province, situated in the Southeastern Anatolia region of Turkey, during June and July 2023. In the power analysis, we determined that we needed to reach at least 156 people with a confidence interval of 90%. We accepted the rate of PTSD in the post-earthquake period as 82.64% by using the sample calculation with an uncertain universe [10]. For the survey, 190 individuals were initially approached, of which 173 volunteers participated. Seventeen individuals (8.95%) declined to participate in the survey. Participant consent was obtained from all participants in the study. The study population comprised earthquake survivors who had experienced the earthquake in the Adıyaman center or its districts on February 6, 2023, and were either breastfeeding or had children aged between 0 and 24 months. Individuals absent in Adıyaman during the earthquake, regardless of whether they stayed in tents or containers, were excluded from the study.

### Data collection

The survey was conducted through face-to-face interviews. A descriptive information form was used as the data collection tool in the study. Participants included earthquake survivors whose homes were either slightly damaged or undamaged and those who had to reside in tents or containers because of their homes being destroyed or severely damaged.

The Turkish version of the PTSD Checklist-Civilian scale utilized in this study was originally developed by Manne et al. in 1998 to assess PTSD symptoms. Neşe Kocabaşoğlu and İlhan Adalet performed the translation into Turkish in 2005, and this translated version underwent rigorous testing for validity and reliability. The Turkish adaptation of the PTSD Checklist-Civilian comprehensively covers all symptoms outlined in the DSM-IV criteria for PTSD and consists of 17 questions. These questions are distributed as follows: five address re-experiencing symptoms, seven relate to avoidance, and five pertain to increased arousal symptoms. Participants’ responses are rated on a scale that spans from “not at all” to “extremely” (ranging from 1 to 5). The aggregated total score can range from a minimum of 17 to a maximum of 85. While the cutoff score commonly employed in numerous studies falls within the range of 24–50 [11], this study adopted a cutoff point of 37, which is more widely accepted in the existing literature. The study revealed that the Cronbach’s alpha value of the scale was 0.821.

BAES, employed in this study, was developed by Hediye Arslan Özkan in 1997 to assess breastfeeding behaviors and attitudes among mothers. This scale consists of 46 items rated on a 5-point Likert scale. Among these, 22 statements were positively scored as 4–3-2–1-0, while the remaining 24 items were reverse-scored as 0–1-2–3-4 to reflect a negative attitude, with “I completely agree” as the highest score. The scale’s total score ranges from a minimum of 0 to a maximum of 184, with 88 points attributed to positive items and 96 to negative items. An increase in the total score indicates a more positive breastfeeding attitude [12]. The study revealed that the Cronbach’s alpha value of the scale was 0.842.

### Statistical analysis

The analyses were conducted using the SPSS package (Statistical Package for Social Sciences; SPSS Inc., Chicago, IL) version 22. Descriptive data were presented as “n” and “%” values for categorical data and mean ± standard deviation (Mean ± SD) values for continuous data. The Chi-square analysis (Pearson Chi-square) was employed to compare categorical variables between groups. The normal distribution of continuous variables was assessed using the Kolmogorov–Smirnov test. The Mann–Whitney U test was utilized to compare paired groups. The Spearman correlation test was applied to examine relationships between continuous variables. A significance level of *p* < 0.05 was considered statistically significant for all analyses.

### Ethical statement

The study was conducted by the principles outlined in the Declaration of Helsinki and received approval from the Non-Interventional Research Ethics Committee of Firat University (protocol code 2023/08–14, dated June 8, 2023). Additionally, institutional permission was obtained from the Adıyaman Provincial Health Directorate (protocol code E-13389845-770-215680146, dated May 16, 2023) to perform the research.

## Results

The average age of the study participants was 31.2 ± 13.2 years (minimum = 19, maximum = 44). Among the participants, 44.5% had completed secondary school education or below, while 55.5% had graduated from high school or higher. Regarding employment, 42.8% were employed, and marital status indicated that 95.4% were married, while 4.6% were widowed. Regarding living conditions, 54.3% of participants resided in tents, 28.9% in containers, and 16.8% at home. 38.2% of participants had lost family members because of the earthquake.

Among the women, 72.3% continued to breastfeed their children. The duration of breastfeeding was reported as 0–6 months for 53.8%, 7–12 months for 32.9%, and 13–24 months for 13.3% of participants. Furthermore, 27.7% of women utilized assistive devices while breastfeeding. A majority, 60.7% of participants, introduced complementary food for their children. The earthquake resulted in reduced breast milk supply for 78.6% of women. Notably, 68.8% of participants had received breastfeeding training.

Regarding PTSD, the average total score for participants was 41.9 ± 17.4%. Among them, 49.7% exhibited symptoms of PTSD. The mean total score for the BAES among women was 86.7 ± 57.2 (Table 1).

**Table 1:** All characteristics of the participants

		Number	%
Age, Mean ± SD		30.0 ± 7.2	
PTSD score, Mean ± SD		41.9 ± 17.4	
BAES score, Mean ± SD		86.7 ± 57.2	
Educational status	Secondary school or below	77	44.5
	High school or higher	96	55.5
Employment status	Employed	74	42.8
	Unemployed	99	57.2
Marital status	Married	165	95.4
	Widowed	8	4.6
Residence place	Tent	94	54.3
	Container	50	28.9
	Home	29	16.8
Presence of death in the family	Yes	66	38.2
	No	107	61.8
Continuing breastfeeding	Yes	125	72.3
	No	48	27.7
Breastfeeding duration	0–6 months	93	53.8
	7–12 months	57	32.9
	13–24 months	23	13.3
Use of assistive devices while breastfeeding	Yes	48	27.7
	No	125	72.3
Complementary food	Yes	105	60.7
	No	68	39.3
Reduced breast milk supply because of earthquake	Yes	136	78.6
	No	37	21.4
Received breastfeeding training status	Yes	119	68.8
	No	54	31.2
Presence of PTSD	Yes	86	49.7
	No	87	50.3

*Abbreviations*: *PTSD* Post-traumatic stress disorder, *BAES* Breastfeeding attitudes of the evaluation scale

The PTSD total score of individuals who graduated from secondary school or below was significantly higher than those who graduated from high school and above (*p* = 0.009). The PTSD scores of employees were considerably lower than those of the unemployed (*p* = 0.006). Both the PTSD score (*p* < 0.001) and the presence of PTSD (*p* = 0.003) among married individuals were significantly lower than those who were widowed. The PTSD score (*p* < 0.001) and the presence of PTSD (*p* < 0.001) among individuals staying in tents were significantly higher than the scores of those staying in other places. Both the PTSD score (*p* < 0.001) and the presence of PTSD (*p* = 0.004) were significantly higher among those who had experienced a family member’s death because of the earthquake. The PTSD score (*p* < 0.001) and the presence of PTSD (*p* < 0.001) were significantly lower among individuals who continued to breastfeed compared to those who did not. Similarly, both the PTSD score (*p* < 0.001) and the presence of PTSD (*p* < 0.001) were significantly higher in those with a breastfeeding duration of 0–6 months compared to those with a breastfeeding duration of 7–24 months (Table 2).

**Table 2:** Comparison of PTSD score and presence of PTSD according to various characteristics of the participants

		PTSD total score		
		Mean ± SD	U	p^*^
Educational status	Secondary school or below	45.8 ± 18.7	-2.604	**0.009**
	High school or higher	38.9 ± 15.7		
Employment status	Employed	38.6 ± 16.6	2.734	**0.006**
	Unemployed	44.4 ± 17.7		
Marital status	Married	40.7 ± 16.7	3.908	** < 0.001**
	Widowed	67.8 ± 9.0		
Residence place	Tent	47.6 ± 17.4	-4.533	** < 0.001**
	Other	35.2 ± 14.9		
Presence of death in the family	Yes	49.5 ± 17.3	-5.011	** < 0.001**
	No	37.2 ± 15.8		
Continuing breastfeeding	Yes	37.0 ± 16.4	5.805	** < 0.001**
	No	54.7 ± 12.9		
Breastfeeding duration	0–6 months	51.4 ± 14.5	-7.705	** < 0.001**
	7–24 months	30.9 ± 13.6		
Use of assistive devices while breastfeeding	Yes	41.2 ± 19.9	-0.141	0.888
	No	42.2 ± 16.4		
Complementary food	Yes	35.8 ± 15.8	5.438	** < 0.001**
	No	51.5 ± 15.4		
Reduced breast milk supply because of earthquake	Yes	46.1 ± 17.3	-5.625	** < 0.001**
	No	26.7 ± 4.3		
Received breastfeeding training status	Yes	32.5 ± 11.0	10.193	** < 0.001**
	No	62.8 ± 8.3		

*Abbreviations*: *PTSD* Post-traumatic stress disorder, *U* Mann–Whitney U test statistic

^*^Mann Whitney U test was applied

The incidence of PTSD was significantly lower (35.4%) in those who used assistive devices during breastfeeding compared to those who did not (55.2%) (*p* = 0.02). Additionally, both the PTSD score (*p* < 0.001) and the presence of PTSD (*p* < 0.001) were significantly lower among those who did not switch to complementary food. Conversely, both the PTSD score (*p* < 0.001) and the presence of PTSD (*p* < 0.001) were significantly higher among those whose milk was reduced because of the earthquake compared to those whose milk did not decrease. Lastly, both the PTSD score (*p* < 0.001) and the presence of PTSD (*p* < 0.001) were significantly lower among those who received breastfeeding education compared to those who did not receive such education (Table 3).

**Table 3:** Comparison of PTSD score and presence of PTSD according to various characteristics of the participants

		Presence of PTSD					
		Number	%	Number	%	χ2	p^**^
Educational status	Secondary school or below	43	55.8	34	44.2	2.088	0.148
	High school or higher	43	44.8	53	55.2		
Employment status	Employed	35	47.3	39	52.7	0.301	0.583
	Unemployed	51	51.5	48	48.5		
Marital status	Married	78	47.3	87	52.7	8.485	**0.003**
	Widowed	8	100.0	0	.0		
Residence place	Tent	61	64.9	33	35.1	18.981	** < 0.001**
	Other	25	31.6	54	68.4		
Presence of death in the family	Yes	42	63.6	24	36.4	8.277	**0.004**
	No	44	41.1	63	58.9		
Continuing breastfeeding	Yes	43	34.4	82	65.6	42.247	** < 0.001**
	No	43	89.6	5	10.4		
Breastfeeding duration	0–6 months	74	79.6	19	20.4	71.723	** < 0.001**
	7–24 months	12	15.0	68	85.0		
Use of assistive devices while breastfeeding	Yes	17	35.4	31	64.6	5.430	**0.02**
	No	69	55.2	56	44.8		
Complementary food	Yes	31	29.5	74	70.5	43.546	** < 0.001**
	No	55	80.9	13	19.1		
Reduced breast milk supply because of earthquake	Yes	86	63.2	50	36.8	46.525	** < 0.001**
	No	0	.0	37	100.0		
Received breastfeeding training status	Yes	32	26.9	87	73.1	79.417	** < 0.001**
	No	54	100,0	0	.0		

*Abbreviations*: *PTSD* Post-traumatic stress disorder, *χ*2 Chi-square test statistic

^**^Chi-square analysis was applied

Individuals who are married (*p* < 0.001), those who do not stay in tents (*p* = 0.001), those who did not experience a death in their families (*p* < 0.001), those who continue to breastfeed (*p* < 0.001), those who breastfeed for 7–24 months (*p* < 0.001), those who switch to supplementary food (*p* < 0.001), those whose milk did not decrease because of the earthquake (*p* < 0.001), and those who received breastfeeding training (*p* < 0.001) exhibited a significantly higher BAES total score (Table 4).

**Table 4:** Comparison of BAES scores according to various characteristics of the participants

		BAES total score		
		Mean ± SD	U	p^*^
Educational status	Secondary school or below	80.5 ± 58.0	1.194	0.233
	High school or higher	91.6 ± 56.3		
Employment status	Employed	93.1 ± 60.1	-1.190	0.234
	Unemployed	81.9 ± 54.7		
Marital status	Married	90.1 ± 56.4	-4.347	** < 0.001**
	Widowed	16.5 ± 5.0		
Residence place	Tent	71.8 ± 54.1	3.466	**0.001**
	Other	104.4 ± 55.9		
Presence of death in the family	Yes	66.8 ± 51.0	3.607	** < 0.001**
	No	98.9 ± 57.6		
Continuing breastfeeding	Yes	111.0 ± 48.7	-10.172	** < 0.001**
	No	23.3 ± 6.3		
Breastfeeding duration	0–6 months	43.8 ± 30.4	10.557	** < 0.001**
	7–24 months	136.5 ± 37.1		
Use of assistive devices while breastfeeding	Yes	104.4 ± 72.2	-1.440	0.150
	No	79.9 ± 48.9		
Complementary food	Yes	111.1 ± 54.8	-7.113	** < 0.001**
	No	49.0 ± 36.8		
Reduced breast milk supply because of earthquake	Yes	70.7 ± 48.2	6.574	** < 0.001**
	No	145.6 ± 49.0		
Received breastfeeding training status	Yes	106.8 ± 56.8	-6.264	** < 0.001**
	No	42.4 ± 22.7		

*Abbreviations*: *BAES* Breastfeeding attitudes of the evaluation scale, *U* Mann–Whitney U test statistic

^*^Mann Whitney U test

A weak positive correlation was identified between age and PTSD score, while a significant weak negative correlation was observed between age and BAES. Furthermore, a significant strong negative correlation was found between the PTSD score and BAES, as illustrated in Table 5.

**Table 5:** Correlation of scale scores

		Age	PTSD total score
PTSD total score	r^a^	**.230**	
	*p*	**.002**	
BAES	r^a^	**-.228**	**-.742**
	*p*	**.003**	**.000**

*Abbreviations*: *PTSD* Post-traumatic stress disorder, *BAES* Breastfeeding attitudes of the evaluation scale

^a^Spearman correlation analysis was used

## Discussion

Postpartum depression and postpartum PTSD can have detrimental effects on the mental and physical well-being of mothers and their infants. In the two-year postpartum breastfeeding period, including the postpartum period, the earthquake, which can cause PTSD alone, makes the situation mentally more difficult for both mother and baby. For this reason, breastfeeding attitudes and behavior, which are closely related to not only the physical but also the mental health of the mother, should be better understood in times of disaster. Because breastfeeding is crucial for the mental health of the mother as well as the physical development of the baby, PTSD, and breastfeeding, which are expected to be closely related to each other, were examined in this study conducted after a major earthquake.

The recommendation for uninterrupted breastfeeding for up to 2 years after birth is well-established [13]. However, in Turkey, the breastfeeding rate beyond 12 months is only 28%, with a median breastfeeding duration of 16.7 months [14]. In the present study, only 13.3% of earthquake survivor mothers breastfed their children for 13–24 months. This finding aligns with research suggesting that breastfeeding tends to cease prematurely after earthquakes [8, 9]. During disasters such as earthquakes, many mobile health teams provide on-site health care to people residing in tents, containers, or houses. These teams should explain to breastfeeding mothers the importance of breastfeeding for their health and the health of their babies, inform them not to cease breastfeeding, and encourage them to breastfeed.

The present study identified a significantly higher PTSD total score (*p* = 0.009, *p* = 0.006) among mothers with lower education and those unemployed. Other studies have also reported higher rates of PTSD among individuals with lower education and unemployment; conflicting results exist, indicating no consistent association between education level, employment status, and PTSD [15]. Lower education and unemployment can be because of many reasons. Therefore, studies involving participants from different geographies and sociocultural structures may yield different results. In addition, results may differ depending on the cause of PTSD.

Furthermore, the present study found both the PTSD score (*p* < 0.001) and the prevalence of PTSD (*p* = 0.003) to be significantly lower among married individuals compared to widows. This trend aligns with the findings of Guo et al. [16] and Kun et al. [17], who noted a higher incidence of post-earthquake PTSD among unmarried or divorced individuals. All these findings suggest that spouses play a crucial role in protecting mothers’ mental health. For this reason, we recommend that spouses of married mothers should also be involved in the treatment and follow-up of PTSD.

We observed a significant positive correlation between staying in tents and higher PTSD scores (*p* < 0.001). This finding aligns with Anwar et al.’s research, which highlighted a more than threefold increase in the risk of PTSD among women residing in tents post-earthquake [18]. However, contrasting findings have also been reported; for instance, Naeem et al. found no association between tent living and PTSD, though they did identify a link with general psychiatric morbidity [19]. The duration of living in a tent after the earthquake, and access to basic needs such as food, water, and toilets may change the incidence of PTSD and other psychiatric morbidity. For this reason, studies may have produced different results from each other.

Breast milk’s positive impact on maternal mental health is well-documented [7]. The present study revealed that both the PTSD score (*p* < 0.001) and the prevalence of PTSD (*p* < 0.001) were lower among mothers who continued breastfeeding. Moreover, both the PTSD score (*p* < 0.001) and the presence of PTSD (*p* < 0.001) were higher in mothers who breastfed for 0–6 months compared to those who breastfed for 7–24 months. These findings suggest breastfeeding may offer protection against post-earthquake PTSD for mothers beyond its established benefits of infant health. Similarly, Garthus-Niegel et al. found a significant increase in PTSD among women who ceased breastfeeding within the first 12 months [20]. It is worth considering that mothers with PTSD might avoid close contact with their infants or resist breastfeeding. Stress-induced cortisol elevation can also impede oxytocin release, reducing breast milk production [21]. In essence, PTSD may contribute to discontinued breastfeeding.

The study revealed that 78.6% of women experienced decreased breast milk because of the earthquake. This finding is consistent with Nouri et al.’s observations of decreased breast milk production after earthquakes [22]. Furthermore, both the PTSD score (*p* < 0.001) and the presence of PTSD (*p* < 0.001) were significantly higher among those with decreased milk supply. This finding raises the possibility that PTSD triggers a perception of inadequate milk supply or, conversely, reduced milk supply exacerbates PTSD. This interplay might contribute to a self-perpetuating cycle.

Our findings underscored the significance of breastfeeding education. Those who received breastfeeding training exhibited higher BAES total scores (*p* < 0.001). Notably, individuals who received breastfeeding education had significantly lower PTSD scores (*p* < 0.001) and a lower prevalence of PTSD (*p* < 0.001) compared to those who did not receive such training. Additionally, a significant negative correlation was observed between PTSD score and BAES. Consistent with our study, İsbir et al. reported that antenatal education was associated with a reduced incidence of postpartum PTSD [23]. These results emphasize the importance of prenatal education about breastfeeding for all pregnant women. Therefore, breastfeeding education should be given to all pregnant women regularly during pregnancy follow-up. Health professionals should also show sensitivity in this regard.

The present study identified a positive correlation between age and PTSD, corroborating Kun et al.’s findings that advanced age is a risk factor for post-earthquake PTSD [17]. However, Dell’Osso et al. reported a higher rate of PTSD among young women compared to older women, with no age-related difference in men [24]. However, since the mother’s age at marriage, number of children, and some other variables may affect PTSD, the studies may have yielded different results.

In line with all these results, politicians should regulate the emergency laws to be implemented after disasters such as earthquakes in a way that gives priority to mothers during the breastfeeding period in terms of nutrition, shelter, and access to health services. In addition, municipalities and the Disaster and Emergency Management Presidency must take all precautions regarding this situation, which is of vital importance for the health of both mother and baby.

The present study has certain limitations. Firstly, we could not distinguish whether the observed cases of PTSD were explicitly related to the postpartum period, were triggered by the earthquake, or resulted from a complex interplay between childbirth and earthquake-related experiences. Moreover, the absence of a comparison group of individuals unaffected by the earthquake could impact the comprehensive understanding of our findings. The difference in the place of residence after the earthquake is another limitation of the study, as it may change the mental structure, behavior, and attitudes of the participants in every subject. Lastly, the timing of our study, conducted in the fifth and sixth months after the quake, may restrict the applicability of our results to earlier or later stages in the aftermath of the earthquake. It would be more appropriate to plan future studies for three periods in the short, medium, and long term after earthquakes or similar disasters, in a way to distinguish whether PTSD is related to delivery or earthquake take into account the participants’ place of residence.

## Conclusion

This study underscores the impact of earthquakes on breastfeeding rates, revealing a decline in breastfeeding among mothers following seismic events. Importantly, breastfeeding emerges as a potential protective factor in post-earthquake PTSD, with breastfeeding education contributing to reduced PTSD presence and severity. The study promotes the spread of knowledge about breastfeeding in disaster management strategies. Moreover, the association between lower education levels, increased unemployment, and higher PTSD rates highlight the need for women’s education and workforce engagement. Additionally, the study underscores the urgency of transitioning earthquake survivors from tents to permanent housing to mitigate heightened PTSD occurrence and promote the well-being of mothers and infants in the aftermath of earthquakes.

## Data Availability

The datasets used and analyzed during the current study are available from the corresponding author on reasonable request.
